# Extensive Cerebral Venous Sinus Thrombosis in a Young Male With Severe Hyperhomocysteinaemia: An Unusual Presentation With Early Diplopia

**DOI:** 10.7759/cureus.102233

**Published:** 2026-01-24

**Authors:** Sooraj Narayan, Balaji Kombde, Atish Komwad

**Affiliations:** 1 Radiodiagnosis, Government Medical College, Latur, Latur, IND

**Keywords:** cerebral venous sinus thrombosis, diplopia, hyperhomocysteinaemia, intracranial hypertension, magnetic resonance venography

## Abstract

Cerebral venous sinus thrombosis (CVST) is a rare cause of stroke, particularly in young adults, and often presents with nonspecific symptoms that may delay diagnosis. Hyperhomocysteinaemia is a recognized but frequently overlooked prothrombotic risk factor. We report a case of a 25-year-old previously healthy male who presented with sudden-onset severe unilateral headache and projectile vomiting. Neuroimaging with magnetic resonance imaging and venography revealed extensive thrombosis involving the superior sagittal, left transverse, and left sigmoid sinuses with cortical veins involvement. Laboratory evaluation showed markedly elevated serum homocysteine levels, with no other identifiable risk factors.

The patient was treated with therapeutic anticoagulation, osmotic agents, and vitamin supplementation for hyperhomocysteinaemia. During treatment, he developed early horizontal diplopia due to left abducens nerve palsy, attributed to evolving intracranial hypertension. Acetazolamide with Fresnel prism eye glasses led to stabilization and gradual improvement of symptoms. This case emphasizes the importance of evaluating metabolic risk factors such as hyperhomocysteinaemia in young patients with CVST and highlights the need for vigilant clinical monitoring for neurological deterioration even after initiation of appropriate therapy.

## Introduction

Cerebral venous sinus thrombosis (CVST) is an uncommon but clinically significant cerebrovascular disorder, accounting for approximately 0.5-1% of all strokes [1]. It predominantly affects young adults and often presents with nonspecific symptoms, most commonly headache, which may lead to delayed diagnosis [2]. Unlike arterial stroke, CVST results from impaired venous outflow, causing venous congestion, cerebral edema, and, in some cases, hemorrhagic infarction [3].

A wide range of acquired and inherited risk factors have been associated with CVST, including infections, dehydration, hormonal factors, and thrombophilic states [3]. Hyperhomocysteinemia is a recognized prothrombotic condition that promotes venous thrombosis through endothelial dysfunction, oxidative stress, and altered coagulation pathways [3]. However, severe elevations may remain undetected unless specifically investigated, particularly in young patients without other apparent risk factors [3-5].

We report a case of extensive multi-sinus CVST in a young male in whom severe hyperhomocysteinaemia was the only identifiable predisposing factor. The case is further notable for the early development of horizontal diplopia during active anticoagulation, highlighting the dynamic clinical course of CVST and the importance of early imaging, metabolic evaluation, and close neurological monitoring [1-7].

## Case presentation

A 25-year-old previously healthy male presented to the emergency department with a sudden-onset, severe unilateral headache associated with multiple episodes of projectile vomiting. He described the pain as the most intense headache he had ever experienced. One week prior to presentation, he had experienced left-sided nasal blockage accompanied by headache, which resolved spontaneously.

There was no history of head trauma, fever, seizures, focal neurological deficits, visual disturbances, altered sensorium, recent infections, or exposure to drugs or toxins. He was a non-smoker, did not consume alcohol, and followed a non-vegetarian diet. There was no personal or family history of thrombotic disorders or systemic illness.

On examination, the patient was alert and oriented, with stable vital signs. Neurological examination revealed no motor, sensory, or cerebellar deficits. Cranial nerve examination was normal at presentation. Systemic examination was unremarkable.

Laboratory investigations revealed a hemoglobin level of 16.5 g/dL with an elevated mean corpuscular volume and mean corpuscular hemoglobin. Serum homocysteine was markedly elevated at 68 µmol/L (Table 1). Serum electrolytes, renal and liver function tests, and coagulation profile were within normal limits.

**Table 1:** Summary of major laboratory findings. DRVVT: dilute Russell’s viper venom time; LS: laboratory specific; ANA: antinuclear antibody; APS: antiphospholipid syndrome

Investigation	Result	Reference Range	Clinical Significance
Serum homocysteine	68.3 µmol/L	5-15 µmol/L	Thrombotic risk; Severe hyperhomocysteinemia
Activated partial thromboplastin time (aPTT, LS)	35.2 s	35.08-43.81 s	Normal
Lupus anticoagulant (DRVVT + mixing study)	Absent	Absent	No evidence of lupus anticoagulant
β2-glycoprotein I IgG antibody	Negative (0.50 U/mL)	<7.0 U/mL	APS serology negative
β2-glycoprotein I IgM antibody	Negative (0.60 U/mL)	<7.0 U/mL	APS serology negative
ANA immunoblot (18-antigen panel)	Negative	Negative	No connective tissue disease serology
Hemoglobin	16.5 g/dL	14-18 g/dL	Normal-high
Total leukocyte count	9,800/mm^3^	4,000-11,000/mm^3^	Normal
Neutrophils	84.5%	45-75%	Neutrophilia
Lymphocytes	10.0%	20-40%	Lymphopenia
Mean corpuscular volume (MCV)	103 fL	76-96 fL	Macrocytosis
Platelet count	217 ×10^3^/mm^3^	150–450 ×10^3^/mm^3^	Normal
Vitamin B12	137 pg/mL	197-771 pg/mL	Suggests impaired homocysteine metabolism

**Figure 1:**
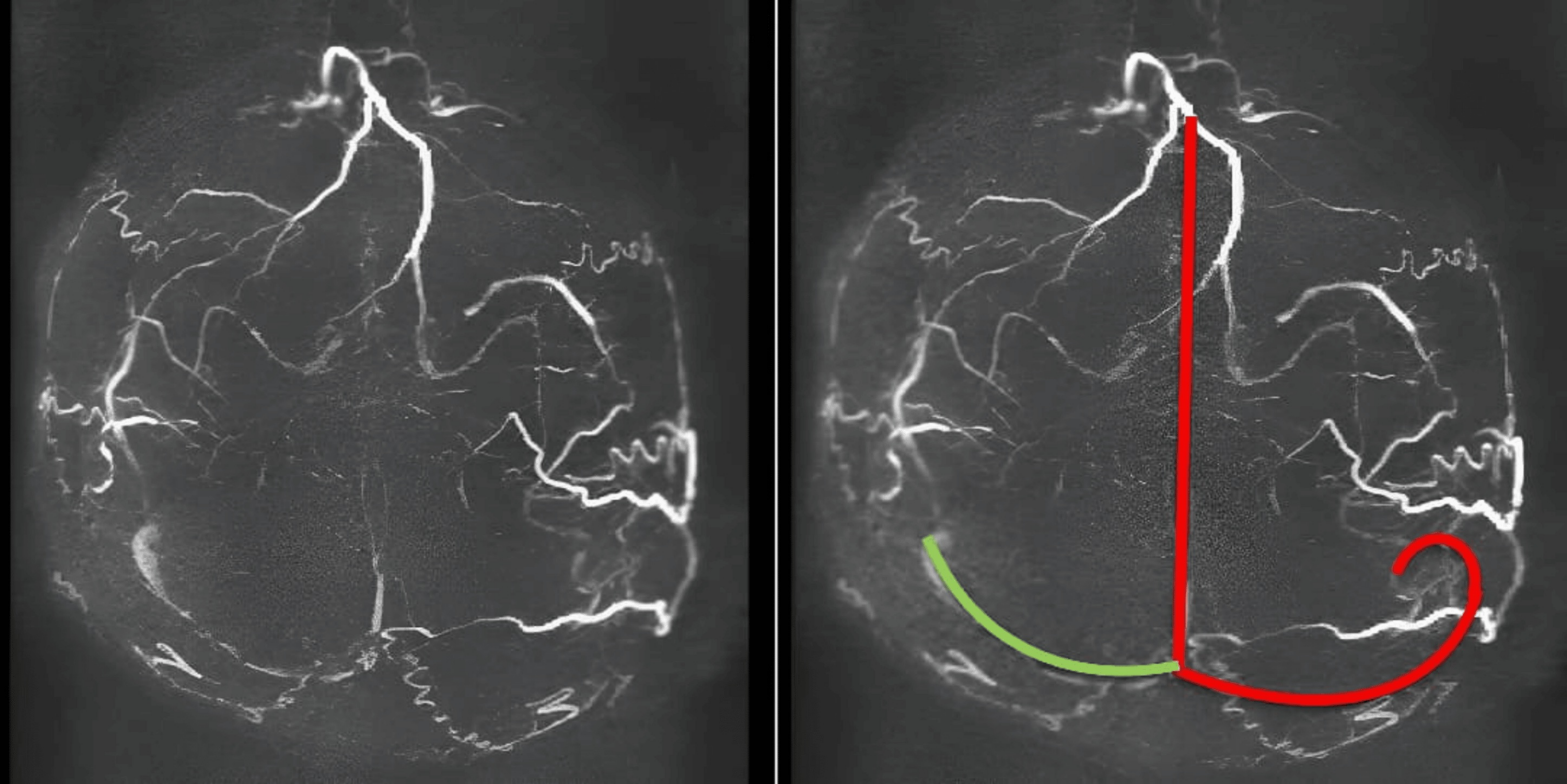
Axial MRV of the brain demonstrating non-visualiztion of superior sagittal, left transverse, and sigmoid sinuses. Note: The red line indicates the normal course of the superior sagittal sinus, left transverse, and left sigmoid sinuses, which are not visualized in the actual MRV image, indicating extensive thrombosis. The green line indicates the course of the right transverse and sigmoid sinuses, that shows normal flow-related signals. MRV: magnetic resonance venography

**Figure 2:**
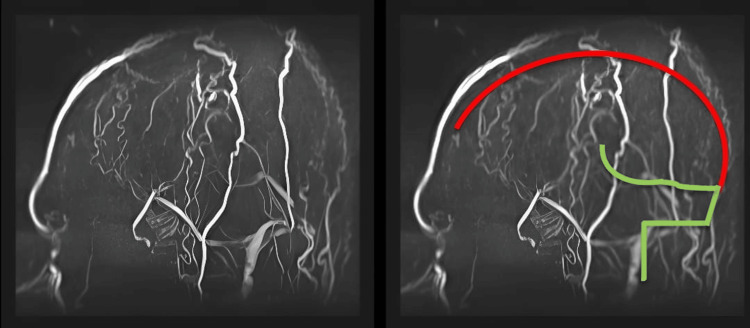
Sagittal MRV of the brain showing non-visualization of superior sagittal sinus. Note: The red line indicates the normal course of the superior sagittal sinus, which is not visualized in the actual MRV image, indicating thrombosis. The green line indicates the normal course of the inferior sagittal sinus and right sigmoid sinus, showing a normal flow-related signal on the MRV image. Serpiginous flow-related signals with a branching pattern are characteristic of superficial scalp arterial branches. MRV: magnetic resonance venography

Given the acute onset and severity of symptoms, magnetic resonance imaging (MRI) of the brain with magnetic resonance venography (MRV) was performed. Imaging demonstrated absent flow within the superior sagittal sinus, along with thrombosis of the left transverse and sigmoid sinuses and involvement of multiple cortical veins, consistent with extensive acute CVST (Figures 1-2). There was no evidence of intracranial hemorrhage or venous infarction. Associated moderate left maxillary sinusitis was noted.

The imaging findings, in conjunction with the markedly elevated serum homocysteine level and absence of other identifiable systemic or thrombophilic risk factors, established the diagnosis of extensive CVST secondary to severe hyperhomocysteinemia.

The patient was promptly initiated on therapeutic anticoagulation with low-molecular-weight heparin to prevent thrombus propagation and facilitate recanalization. Intravenous mannitol was administered to manage intracranial pressure, and supportive measures, including adequate hydration, were ensured. Concurrently, homocysteine-lowering therapy was started, comprising folic acid, vitamin B6, and vitamin B12 supplementation.

**Figure 3:**
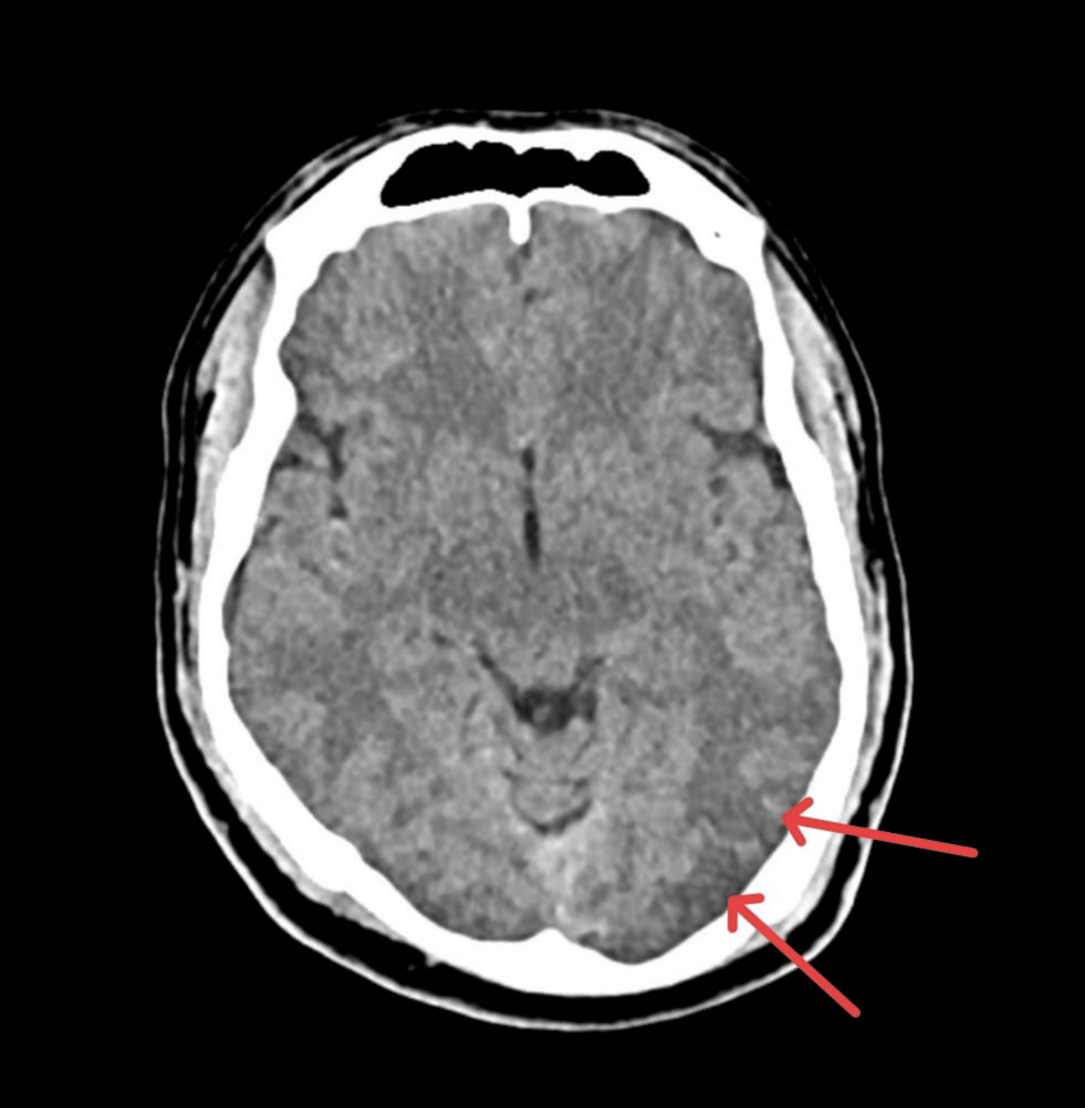
Axial CT of the brain showing subtle cerebral oedema in the left parieto-temporal lobe (red arrows). CT: computed tomography

During the first few days of treatment, the patient developed early-onset horizontal diplopia due to left abducens nerve palsy, characterized by esotropia and limitation of abduction in the left eye, indicative of evolving intracranial hypertension. A computed tomography (CT) scan of the brain confirmed persistent venous thrombosis with subtle parieto-temporal edema, without hemorrhage (Figure 3).

**Figure 4:**
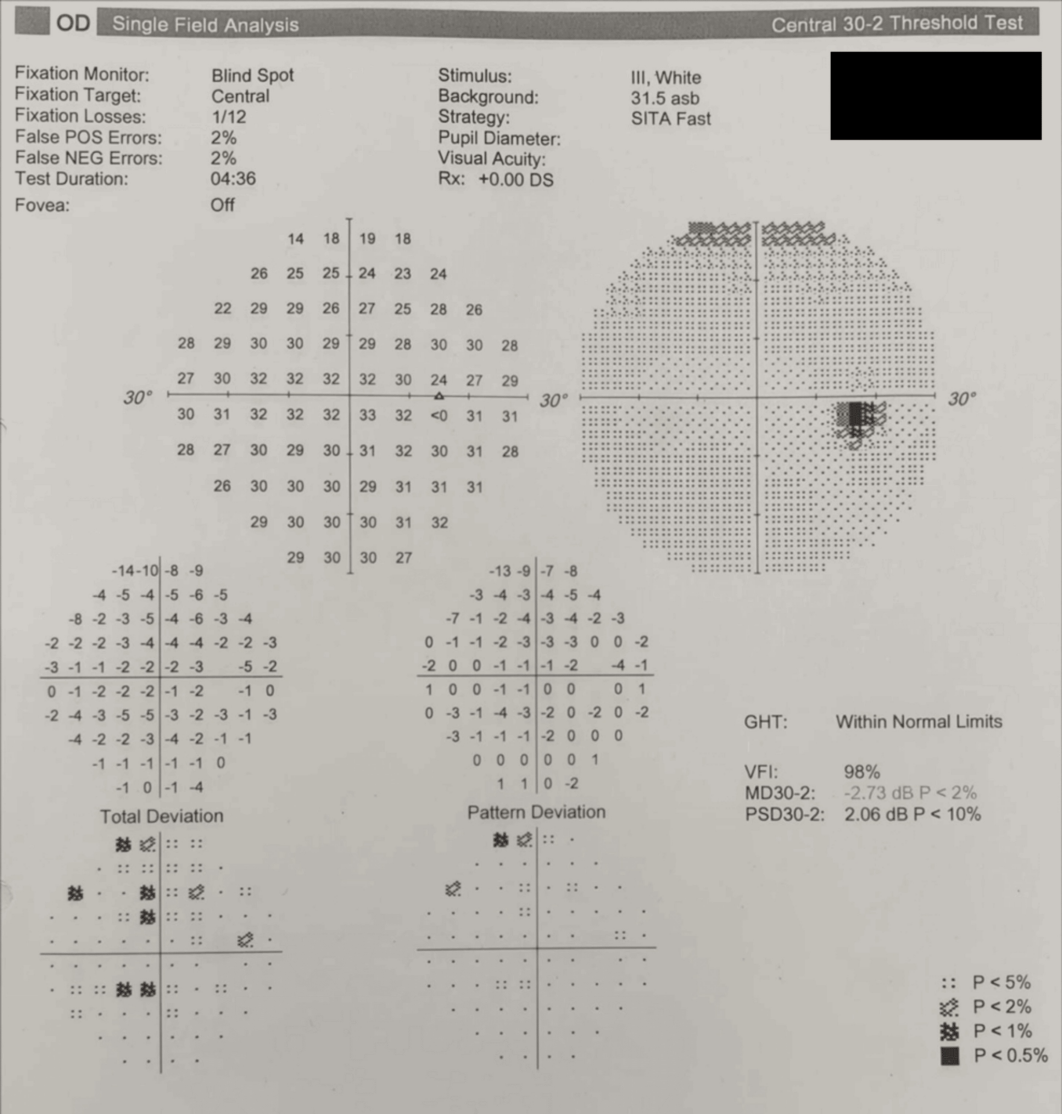
Automated static perimetry (Humphrey Visual Field 30-2) of the right eye demonstrating the absence of a significantly enlarged blind spot.

**Figure 5:**
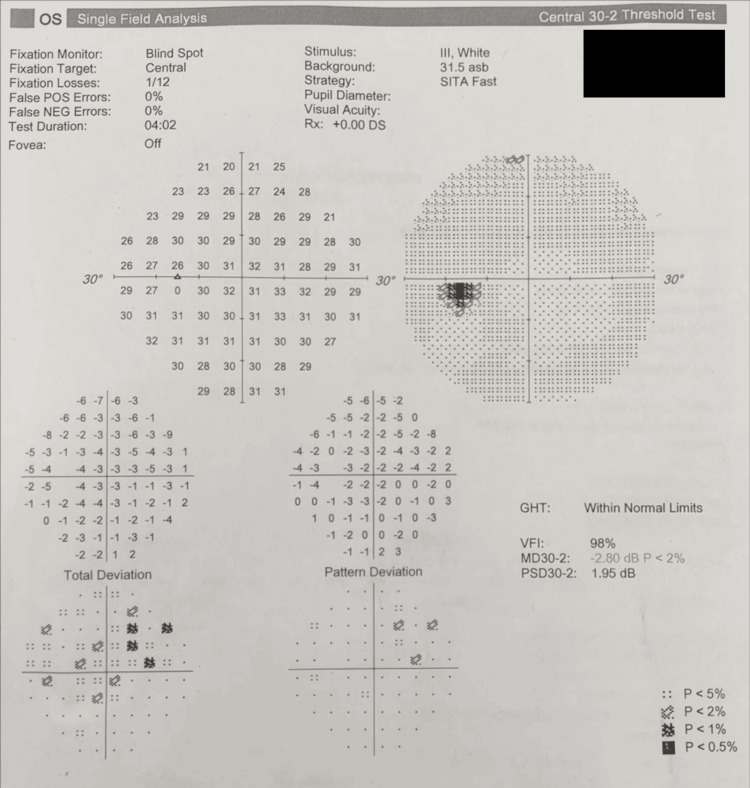
Automated static perimetry (Humphrey Visual Field 30-2) of the left eye demonstrating the absence of a significantly enlarged blind spot.

Fundoscopy and perimetry were normal (Figures 4-5).

Acetazolamide was added to manage intracranial pressure, and Fresnel prism glasses were prescribed for symptomatic relief of diplopia.

On the same day of treatment, he also developed carpopedal spasm and slurring of speech, which was sudden in onset, with positive Chvostek and Trousseau signs owing to low serum calcium levels, for which immediate intravenous administration of calcium gluconate was given that gradually stabilized the patient.

He was transitioned to long-term oral anticoagulation for secondary prevention and discharged with close neurology follow-up. At discharge, he was neurologically stable, ambulatory, and independent in activities of daily living. On outpatient follow-up, the patient reported continued improvement in visual symptoms, with near-complete resolution of diplopia. No recurrent headaches, seizures, or focal neurological deficits were noted. Ongoing neurological follow-up and repeat neuroimaging were planned to assess venous recanalization and to monitor for recurrence.

## Discussion

CVST is an uncommon but clinically significant cause of stroke, accounting for approximately 0.5-1% of all cerebrovascular events and predominantly affecting young adults. The clinical presentation is highly variable, with headache being the most frequent symptom, often mimicking subarachnoid hemorrhage or intracranial space-occupying lesions. This heterogeneity contributes to diagnostic delay, particularly when conventional vascular risk factors are absent. Early use of MRI with MRV remains the diagnostic modality of choice and is critical when initial CT is unrevealing [1]. The latter should be differentiated from other disorders with similar clinical symptoms (Table 2).

**Table 2:** Differential diagnoses considered. Credits: The authors.

Condition	Key Clinical Features	Imaging Findings	Differentiating Features
Cerebral venous sinus thrombosis	Headache, seizures, focal neurological deficits, papilledema, diplopia	MRI brain with MR venography (MRV) showing a filling defect or absent flow in the venous sinuses	Venous territory involvement; hemorrhagic infarcts may be present
Idiopathic intracranial hypertension	Headache, papilledema, sixth nerve palsy, usually in young females	Normal MRI/MRV or transverse sinus narrowing without thrombosis	No venous sinus thrombosis; normal brain parenchyma
Arterial ischemic stroke	Sudden onset of focal neurological deficit	Infarct in an arterial territory on MRI	Lesions confined to arterial distribution; MRV normal
Subarachnoid hemorrhage	Sudden severe “thunderclap” headache, neck stiffness	CT scan shows subarachnoid blood	Acute onset; no venous sinus involvement
Intracranial space-occupying lesion	Progressive headache, seizures, focal deficits	Mass lesion with surrounding edema and contrast enhancement	Gradual progression; focal mass effect
Meningitis/encephalitis	Fever, headache, altered sensorium, neck stiffness	Meningeal enhancement on MRI; abnormal CSF	Infective features predominate
Migraine with aura	Recurrent episodic headache with visual or sensory aura	Normal neuroimaging	Fully reversible symptoms; no raised intracranial pressure
Posterior reversible encephalopathy syndrome	Headache, seizures, visual disturbances	Symmetric posterior white-matter edema on MRI	Associated with hypertension; venous sinuses patent

Hyperhomocysteinaemia represents a well-established but frequently under-recognized prothrombotic condition in CVST. Homocysteine is a sulfur-containing amino acid generated during methionine metabolism and is regulated through remethylation and transsulfuration pathways that depend on folate, vitamin B_12_, and vitamin B_6_ as essential cofactors. Disruption of these pathways leads to elevated circulating homocysteine levels, which exert multiple deleterious vascular effects [2].

At a biochemical level, elevated homocysteine promotes thrombosis through endothelial dysfunction mediated by oxidative stress, reduced nitric oxide bioavailability, increased expression of tissue factor, enhanced platelet activation, and inhibition of anticoagulant mechanisms such as thrombomodulin-dependent protein C activation [2]. These effects collectively create a prothrombotic milieu that predisposes to venous thrombosis. Severe elevations (>50 µmol/L), as observed in this patient, have been strongly associated with extensive venous involvement and increased thrombotic burden [3].

Several studies support the association between hyperhomocysteinaemia and CVST. Kalita et al. demonstrated a high prevalence of hyperhomocysteinaemia among Indian patients with CVST, frequently in association with vitamin B_12_ deficiency and methylenetetrahydrofolate reductase (MTHFR) gene polymorphisms, particularly the C677T variant [3]. These genetic variants reduce enzyme activity, impair folate metabolism, and further elevate homocysteine levels, thereby amplifying thrombotic risk. Similar associations between MTHFR polymorphisms, elevated homocysteine, and CVST have been reported in additional case series and genetic studies [3,4].

Familial and inherited forms of hyperhomocysteinaemia have also been linked to severe thrombotic manifestations, including combined cerebral venous thrombosis and pulmonary embolism, underscoring the systemic thrombotic potential of this metabolic abnormality [5]. These findings highlight the importance of routine metabolic and nutritional evaluation in young patients with CVST, even when no other thrombophilic factors are identified.

The early development of horizontal diplopia during therapeutic anticoagulation represents a notable and clinically important feature of this case. Diplopia in CVST most commonly results from raised intracranial pressure, causing abducens nerve palsy, owing to the nerve’s long intracranial course and susceptibility to stretch at the clivus. In this patient, evolving venous congestion and parieto-temporal edema likely contributed to increased intracranial pressure despite appropriate anticoagulation. This phenomenon has been documented in previous reports describing sixth nerve palsies and papilledema in CVST, even in the absence of classical headache features [6].

This observation emphasizes that neurological deterioration can occur during the acute phase of treatment and should prompt repeat neuroimaging and escalation of intracranial pressure management rather than premature attribution to treatment failure.

Extensive involvement of the superior sagittal sinus, transverse sinus, sigmoid sinus, and cortical veins is uncommon and is associated with a higher risk of complications, including venous infarction, hemorrhage, and persistent intracranial hypertension. Anticoagulation remains the cornerstone of treatment and is recommended even in the presence of venous infarction, as it prevents thrombus propagation and facilitates recanalization [1]. Adjunctive therapies, such as osmotic agents and acetazolamide, play a crucial role in managing raised intracranial pressure in selected patients.

Long-term management must focus on the correction of underlying metabolic abnormalities. In patients with hyperhomocysteinaemia, sustained vitamin supplementation and evaluation for nutritional or genetic causes are essential to reduce recurrence risk. Regular clinical follow-up and interval neuroimaging are recommended to assess venous recanalization and guide the duration of anticoagulation [1-7].

## Conclusions

CVST should be considered in young adults presenting with acute or subacute severe headache, even in the absence of conventional vascular risk factors. This case highlights severe hyperhomocysteinaemia as a clinically significant and potentially isolated prothrombotic factor capable of causing extensive multi-sinus involvement. Recognition of metabolic contributors is crucial, as hyperhomocysteinaemia represents a modifiable risk factor with implications for both acute management and long-term prevention.

The occurrence of early diplopia during therapeutic anticoagulation underscores the dynamic clinical course of CVST and the possibility of neurological deterioration despite appropriate treatment. This emphasizes the need for close neurological surveillance, repeat neuroimaging when new deficits emerge, and timely management of raised intracranial pressure. Comprehensive evaluation, prompt anticoagulation, targeted correction of underlying metabolic abnormalities, and structured follow-up are essential to optimize outcomes and reduce the risk of recurrence in patients with CVST.
